# Exposure to excess insulin (glargine) induces type 2 diabetes mellitus in mice fed on a chow diet

**DOI:** 10.1530/JOE-14-0117

**Published:** 2014-06

**Authors:** Xuefeng Yang, Shuang Mei, Haihua Gu, Huailan Guo, Longying Zha, Junwei Cai, Xuefeng Li, Zhenqi Liu, Wenhong Cao

**Affiliations:** 1 Department of Nutrition Gillings School of Global Public Health, Nutrition Research Institute (NRI) at Kannapolis, The University of North Carolina at Chapel Hill Chapel Hill, North Carolina, 27559 USA; 2 Department of Nutrition and Food Hygiene Tongji Medical College, Huazhong University of Science and Technology Wuhan, Hubei, 430030 People's Republic of China; 3 Department of Preventive Medicine Hubei University of Medicine Shiyan, Hubei, 442000 People's Republic of China; 4 Department of Nutrition and Food Hygiene School of Public Health and Tropical Medicine, Southern Medical University Guangzhou, 510515 People's Republic of China; 5 Department of Medicine Tai He Hospital, Hubei University of Medicine Shiyan, Hubei, 442000 People's Republic of China; 6 Department of Medicine (Endocrinology) University of Virginia Health System Charlottesville, Virginia, 22908 USA; 7 Department of Medicine (Endocrinology and Metabolism) Duke University School of Medicine Durham, North Carolina, 27705 USA

**Keywords:** insulin resistance, insulin, glargine, diabetes, cholesterol synthesis, T2DM

## Abstract

We have previously shown that insulin plays an important role in the nutrient-induced insulin resistance. In this study, we tested the hypothesis that chronic exposure to excess long-acting insulin (glargine) can cause typical type 2 diabetes mellitus (T2DM) in normal mice fed on a chow diet. C57BL/6 mice were treated with glargine once a day for 8 weeks, followed by evaluations of food intake, body weight, blood levels of glucose, insulin, lipids, and cytokines, insulin signaling, histology of pancreas, ectopic fat accumulation, oxidative stress level, and cholesterol content in mitochondria in tissues. Cholesterol content in mitochondria and its association with oxidative stress in cultured hepatocytes and β-cells were also examined. Results show that chronic exposure to glargine caused insulin resistance, hyperinsulinemia, and relative insulin deficiency (T2DM). Treatment with excess glargine led to loss of pancreatic islets, ectopic fat accumulation in liver, oxidative stress in liver and pancreas, and increased cholesterol content in mitochondria of liver and pancreas. Prolonged exposure of cultured primary hepatocytes and HIT-TI5 β-cells to insulin induced oxidative stress in a cholesterol synthesis-dependent manner. Together, our results show that chronic exposure to excess insulin can induce typical T2DM in normal mice fed on a chow diet.

## Introduction

Type 2 diabetes mellitus (T2DM) has become a major health and economic burden to industrialized societies. Its negative impact on the general health and economy is still rapidly increasing due to the continuing industrialization. It is very clear that the root of T2DM, in most cases, is the excess calories due to overeating and/or lack of physical activity. However, the exact mechanism by which excess calories are converted into T2DM remains unestablished.

T2DM is composed of three key components: insulin resistance, hyperinsulinemia, and relative insulin insufficiency. Relative insulin insufficiency (overt T2DM) typically occurs over 10 years after insulin resistance and hyperinsulinemia have been established (Kendall *et al*. 2009). In other words, insulin resistance/hyperinsulinemia is a precursor of T2DM. It is still highly debatable as to how nutrients cause insulin resistance/hyperinsulinemia and then T2DM. Inflammation, endoplasmic reticulum stress, and mitochondrion-derived oxidative stress have all been shown to be involved in the development of insulin resistance/hyperinsulinemia (Hotamisligil 2010, Samuel *et al*. 2010, Gregor & Hotamisligil 2011). The presence of these multiple mechanisms itself demonstrates that the primary mechanism of insulin resistance has not been established. The primary cause or the original root of insulin resistance and T2DM is ‘excess calories’, which derive from three main dietary components: glucose/carbohydrates, fat/fatty acids, and proteins/amino acids. Thus, we have recently addressed several simple questions. First, does glucose itself cause insulin resistance in the absence of insulin? Our results show that animals with untreated hyperglycemia in type 1 diabetes mellitus (T1DM) respond to acute insulin challenge much better than the diabetic animals treated with insulin, suggesting that hyperglycemia itself does not cause insulin resistance but treatment of T1DM with insulin does (Liu *et al*. 2009*a*). In the absence of insulin, a high level of glucose does not cause insulin resistance in cultured hepatocytes either (Liu *et al*. 2009*a*). Secondly, does fat itself cause insulin resistance without insulin? Our results have shown that induction of insulin resistance by fats from a high-fat diet (HFD) is prevented when insulin secretion is disrupted with streptozotocin (Ning *et al*. 2011), suggesting that dietary fat itself does not cause insulin resistance without insulin. In the absence of insulin or when insulin signaling is blunted, free fatty acids (FFA) do not cause insulin resistance in cultured hepatocytes (Ning *et al*. 2011). Thirdly, does supplementation of amino acids necessarily cause insulin resistance? Results from others and us show that supplementation of amino acids such as leucine to animals fed on a HFD increases insulin sensitivity rather than causing insulin resistance (Zhang *et al*. 2007, Guo *et al*. 2010, Yang *et al*. 2013). Therefore, macronutrients themselves do not cause insulin resistance without insulin. In other words, insulin plays an essential role in converting nutrients into insulin resistance and then T2DM. We have previously shown that blunting insulin signaling can actually prevent the HFD-induced insulin resistance/hyperinsulinemia (Liu *et al*. 2009*b*). However, it is still unknown whether continuous exposure to excess exogenous insulin similar to that observed in subjects with insulin resistance and hyperinsulinemia can cause T2DM in normal mice fed on a chow diet. In this study, we addressed this issue and found that chronic exposure to excess insulin caused insulin resistance, hyperinsulinemia, and relative insulin deficiency, i.e., T2DM, in mice fed on a chow diet.

## Materials and methods

### Reagents and antibodies

Cholesterol, simvastatin, and DCF-DA were purchased from Sigma. Antibodies against phosphorylated Akt or total Akt, insulin receptor, serine^636^- or tyrosine^612^-phosphorylated insulin receptor substrate 1 (IRS1) were obtained from Cell Signaling Technologies, Inc. (Beverly, MA, USA). Antibodies against β-actin, mouse immunoglobulin G (IgG), and rabbit IgG were purchased from Sigma. Amplex red cholesterol assay kit was purchased from Invitrogen. The kit for measuring levels of glutathione (GSH) and glutathione disulfide (GSSG) was purchased from OxisResearch (Foster City, CA, USA).

### Animal experiments

C57BL/6 (B6) mice (male, 6–9-week old) from Charles River were treated with either saline or a long- and slow-acting insulin reagent, glargine (50 Unit/kg body weight, s.c. injection, once a day), for 8 weeks. The dose of glargine was determined according to our previous experiments (Liu *et al*. 2009*a*) and transgenic expression of human insulin gene (32 times of normal insulin level) in animals determined by Marban *et al*. (1989). Mice were housed individually at the regular room temperature, allowed to feed on the regular chow diet (Prolab RMH 3000) *ad libitum*, and kept at 12 h light:12 h darkness cycles. The weekly measurements of blood glucose level were performed after an 8 h fast with a glucose meter (Abbott Diabetes Care, Inc.) with a little drop of tail blood. After an 8 h fast at the end of the 8-week experiment, mice were sedated with pentobarbital (50 mg/kg body weight) and killed by cervical dislocation, and mitochondria from liver, gastrocnemius, and pancreas were isolated as described previously. Mitochondrial cholesterol was quantified. All animal studies were approved by the Institutional Animal Care and Use Committee of The University of North Carolina at Chapel Hill and fully complied with the guidance from the National Institutes of Health.

### Culture of HIT-TI5 hamster pancreatic insulinoma cells

HIT-T15 hamster pancreatic insulinoma cells were purchased from ATCC (Manasas, VA, USA) and cultured in F12K medium containing 10% horse serum, 2.5% fetal bovine serum (FBS), 100 U/ml penicillin, and 100 μg/ml streptomycin at 37 °C in an atmosphere of 5% CO_2_. HIT-T15 cells (2×10^4^ cells/well) were seeded in a 96-well plate and pre-incubated for 24 h before any experimentation.

### Measurements of serum cytokines, hormones, and lipids

Serum triglyceride (TG) and FFA were measured with kits purchased from Cayman Chemical (Ann Arbor, MI, USA) (Cat No: 10010303 and 700310). Leptin, adiponectin, and tumor necrosis factor α (TNFα) were measured with kits obtained from Abcam (Cambridge, UK) (Cat No: ab100718, ab108785, and ab46105). Endogenous insulin and growth hormone were detected with ELISA kits purchased from Millipore (Billerica, MA, USA) (Cat No: EZRMI-13K and EZRMGH-45K).

### Preparation and culture of primary hepatocytes

Mouse primary hepatocytes were isolated from male B6 mice (6–9 weeks) as we described previously (Cao *et al*. 2005, Xiong *et al*. 2006, Collins *et al*. 2007, Liu *et al*. 2009*c*). Viable hepatocytes were suspended in Williams' E medium (Invitrogen) containing antibiotics (50 μg/ml penicillin, 50 μg/ml streptomycin, and 100 μg/ml neomycin). Cells were seeded at a density of 3.5×10^5^ cells per 100 mm Petri dish (BD Falcon) pre-coated with 0.02% collagen type I.

### Measurement of peroxidized lipids

Levels of peroxidized lipids were indirectly determined by measuring the levels of malondialdehyde (MDA), a byproduct of lipid peroxidation with a commercialized kit (Northwest Life Science Specialties, Vancouver, WA, USA) as described previously (Aust *et al*. 1985). The level of MDA was measured by quantifying the absorbance at 532 nm.

### Measurement of MnSOD activity

Manganese superoxide dismutase (MnSOD) activity was determined using a commercially available kit (Cayman Chemical Company) (Papazzo *et al*. 2011). Briefly, xanthine oxidase and hypoxanthine were used to generate superoxide radicals that were then detected by tetrazolium salt and quantified at 540 nm with a microplate reader (Cell Biolabs, USA). One unit of SOD was defined as the amount of enzyme required to inhibit the dismutation of the superoxide radical by 50%. Potassium cyanide (KCN, 1 mM)) was used to inhibit the Cu- and Zn-SOD.

### Histological examination of pancreas

Pancreatic slices (5 mm thickness) were fixed in 10% neutral-buffered formalin/Bouin's fluid overnight. All the tissue samples were subjected to standard processing and staining procedures. Paraffin-embedded sections of 5-micron were stained with hematoxylin and eosin, and examined under a light microscope (Leitz Diaplan, USA).

### Measurement of GSH/GSSG

Levels of GSH and GSSG in tissue lysates were determined with a kit purchased from OXIS International, Inc. (Foster City, CA, USA) and normalized to protein levels. Measurements of GSH and GSSG and calculation of the GSH:GSSG ratio were performed in the following four steps: i) determination of the reaction rate by time; ii) construction of calibration curve; iii) calculation of concentrations; and iv) calculation of the GSH:GSSG ratio as GSH:GSSG ratio=(GSH−2GSSG)/GSSG.

### RNA extraction and real-time PCR

Total RNAs were extracted from tissues with an RNeasy Mini Kit (Qiagen) and reverse transcribed into cDNAs, which were quantified by TaqMan Real-time PCR with specific probes and primers and normalized to levels of m36B4.

### Preparation of mitochondria

To isolate mitochondria, tissue samples were collected promptly. Mitochondria were isolated as described previously (Palmer *et al*. 1977). Tissues were then lysed with 1 ml ice-cold lysis buffer by pipetting up and down several times, incubated for 10 min on ice on a shaker, and then precipitated by centrifugation (at 1000 ***g***, for 10 min). Supernatants were removed and pellets were resuspended in 1.5 ml MSE buffer containing 225 mM mannitol, 75 mM sucrose, 1 mM EGTA, 5 mM HEPES, pH 7.4, and 1 mg/ml BSA, followed by centrifugation at 1000 ***g*** for 3 min. Then supernatants were transferred to a clean 1.5 ml tube, followed by centrifugation at 6000 ***g*** for 10 min. Supernatants were removed and pellets, mitochondria, were resuspended in 1 ml MSE.

### Measurement of cholesterol

Lipids in isolated mitochondria were extracted with isopropanol. Free cholesterol was quantified using the Amplex red cholesterol assay kit. Meanwhile, protein levels in the same samples were measured with standard Bio-Rad assays.

### Immunoblotting

Proteins (30 μg) were denatured at 95 °C for 5 min in a loading buffer (60 mM Tris, 2.5% SDS, 10% glycerol, 5% mercaptoethanol, and 0.01% bromophenol blue) and subjected to 10% SDS–PAGE. Proteins in gels were transferred onto PVDF membranes and blocked with TBS containing 0.05% Tween 20 (TBS-T) and 5% non-fat milk for 1 h. After being washed with TBS-T, membranes were probed with specific first antibodies against target proteins (rabbit) or β-actin (mouse) (1:1000) overnight at 4 °C. Membranes were then washed with TBS-T and incubated with polyclonal secondary antibodies (1:5000) for 1 h at room temperature. After three washes with TBS-T, membranes were treated with ECF substrates (GE Healthcare, Pittsburgh, PA, USA). Fluorescent bands were visualized and then quantified by densitometry analysis using the ImageQuant version 5.2 software obtained from GE Healthcare.

### Measurement of ROS production from cultured cells

Intracellular reactive oxygen species (ROS) was detected using fluorescent DCF-DA as described previously (Palmer *et al*. 1977, D'Alessandro *et al*. 2011). In brief, the non-fluorescent dye freely permeates into cells, where it is de-esterified to form ionized free acid (dichlorofluorescein) and reacts with ROS to form fluorescent 2′,7′-dichlorofluorescein (DCF). Cells were washed thrice with PBS and then loaded with 20 μM H_2_DCF-DA for 30 min at 37 °C. The fluorescence of DCF was analyzed using a plate reader (Spectramax Gemini EM, Molecular Devices) at the excitation and emission wavelengths of 490 and 530 nm respectively.

### Statistical analysis

Data are presented as mean±s.d./s.e.m. Data were compared by Student's *t*-test with GraphPad Prism version 4.0 for Windows (San Diego, CA, USA). Differences at values of *P*<0.05 were considered significant.

## Results

### Chronic exposure to excess insulin increases white fat:body weight ratio

**Figure 1:**
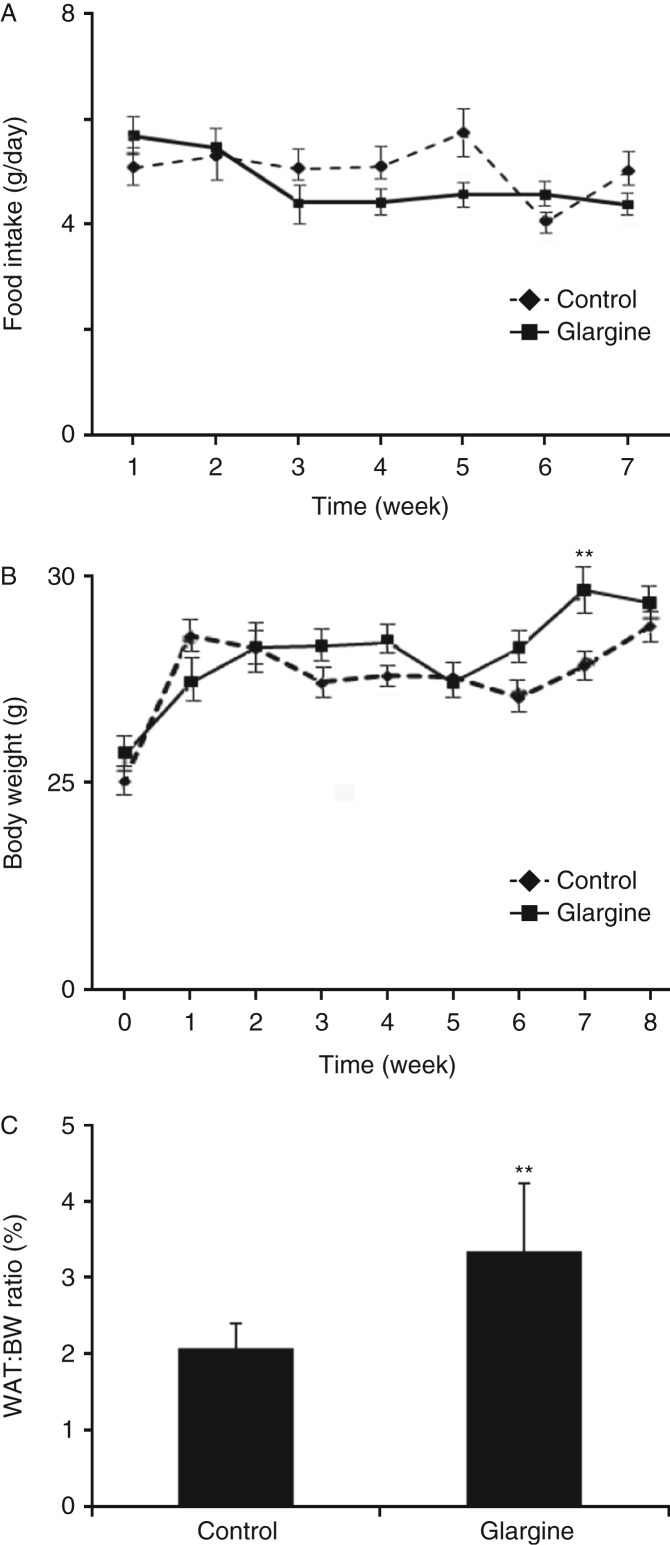
Chronic exposure to excess insulin (glargine) increases the ratio of white fat to body weight without obviously altering food intake and body weight. C57CL/6 (B6) mice were treated with either glargine or saline daily for 8 weeks. Food intake (A) and body weight (B) were measured weekly. Epididymis fat tissue was collected at the end of the experiment. (C) The ratio of epididymis fat to body weight was calculated. Results represent mean±s.d. of six to eight mice/group. **P<0.01 vs control.

During the 8-week treatment with glargine, food intake and body weight were measured weekly. As shown in Fig. 1A, mice treated with glargine consumed slightly more diet at the first week and then tended to consume slightly less diet when compared with control mice although the changes did not reach a statistical significance. Mice treated with glargine gained slightly more body weight than control mice although the gain reached a statistical significance only on week 7 (Fig. 1B). However, the percentage of epididymal fat (white adipose tissue, WAT) over the whole-body weight was significantly increased in mice treated with glargine. Treatment with glargine did not alter plasma levels of leptin, adiponectin, TNFα, TG, and FFA but increased plasma level of cholesterol (Table 1). These results together demonstrate that chronic exposure to glargine increases WAT and blood cholesterol level.

### Chronic exposure to excess insulin induces T2DM in normal mice fed on a chow diet

**Figure 2:**
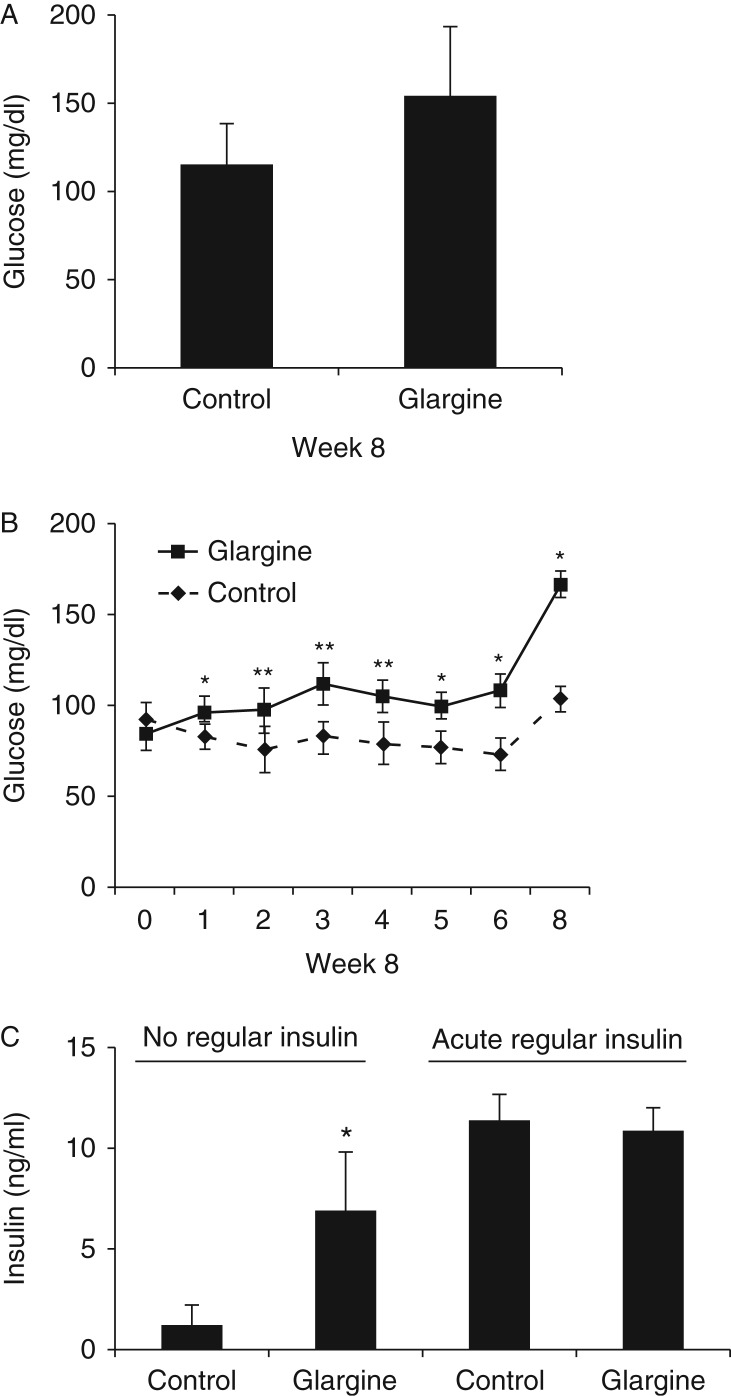
Chronic exposure to excess insulin (glargine) induces T2DM in normal mice fed on a chow diet. After an 8 h fast, blood glucose (A and B) and serum insulin (C) of mice described in Fig. 1 were measured. Some animals were treated with regular human insulin for 15 min (0.75 Unit/kg body weight, i.p.) after an 8 h fast, followed by measurement of serum insulin. Results represent mean±s.d. of six to eight animals/group. *P<0.05 vs control. **P<0.01 vs control.

Fasting blood glucose levels were measured weekly during the experiment. Signs of hypoglycemia such as poor grooming and shivering were closely monitored, particularly during the first 2 days of the glargine treatment. No sign of hypoglycemia was ever noted. As shown in Fig. 2A and B, fasting blood glucose level in mice treated with glargine started to increase on week 1 and significantly increased (150 mg/dl) on week 8. Fasting level of plasma endogenous insulin measured was significantly increased by glargine treatment (Fig. 2C). It should be noted that the antibodies against insulin used in the ELISAs, in this study, recognized only the regular insulin but not glargine. Hence, the insulin detected here was endogenous or regular insulin. Furthermore, administration of regular insulin during the insulin tolerance increased the blood level of regular insulin equally in mice with or without glargine treatment (Fig. 2C). The increase in levels of both fasting blood glucose and endogenous insulin established the notion that chronic exposure to excess insulin induced insulin resistance in animals treated with glargine. These results together demonstrate that chronic exposure to excess insulin/glargine induced T2DM characterized by insulin resistance, hyperinsulinemia, and relative insulin insufficiency.

### Chronic exposure to excess insulin induces insulin resistance mainly in liver

**Figure 3:**
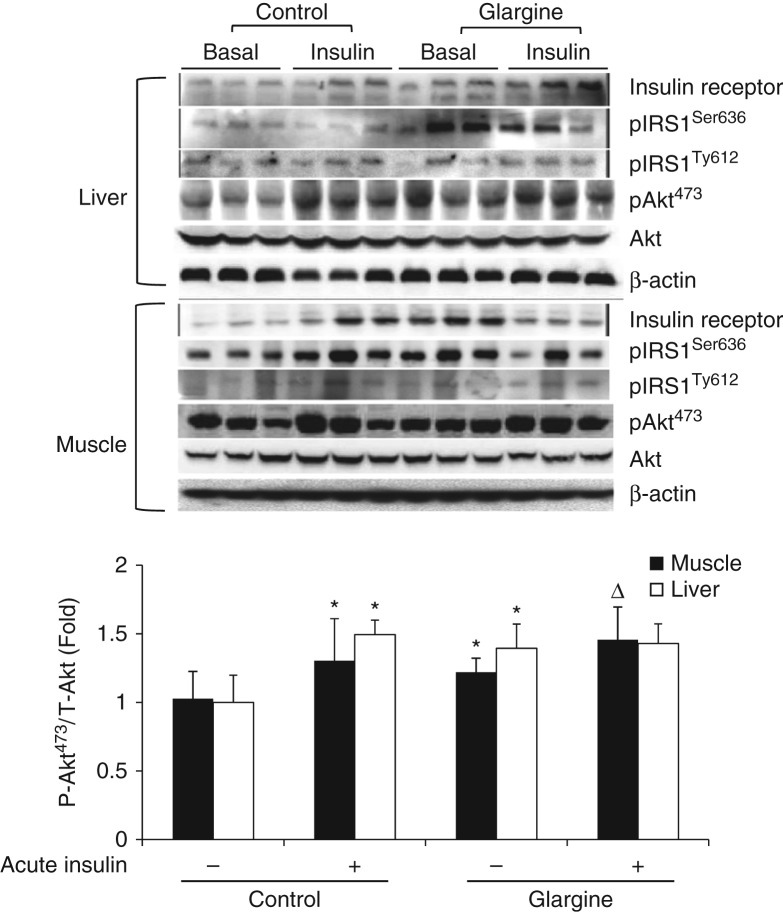
Chronic exposure to insulin (glargine) induces tissue-specific insulin resistance in normal mice fed on a chow diet. After an 8 h fast, some mice described in Fig. 1 as noted were treated with regular human insulin (0.75 Unit/kg body weight, i.p.) for 15 min, followed by measuring levels of insulin receptor, IRS1 serine phosphorylation, IRS1 tyrosine phosphorylation, Akt phosphorylation, total Akt, and β-actin using the immunoblotting technique with specific antibodies. Akt phosphorylation was quantified and presented as mean±s.d. of three animals. *P<0.05 vs no acute insulin treatment in control. ^Δ^ *P*<0.05 vs no acute insulin treatment in animals treated with glargine.

To determine the effect of chronic exposure to excess insulin on insulin sensitivity, levels of insulin receptor, serine and tyrosine phosphorylations of IRS1, and Akt phosphorylation in liver and skeletal muscle (gastrocnemius) were determined using the immunoblotting technique. As shown in Fig. 3A, levels of insulin receptor and tyrosine phosphorylation of IRS1 were not altered obviously by glargine treatment in both liver and gastrocnemius. IRS1 serine phosphorylation was increased in both liver and gastrocnemius, but much more dramatically in liver, by glargine. Acute insulin challenge for 15 min did not alter serine and tyrosine phosphorylations of IRS1 in liver or gastrocnemius obviously. However, acute insulin challenge elevated Akt phosphorylation in both liver and gastrocnemius in control mice (Fig. 3A and B). Treatment with glargine increased the basal level of Akt phosphorylation in both liver and gastrocnemius. Acute challenge with insulin stimulated Akt phosphorylation in gastrocnemius but not in liver of mice treated with glargine (Fig. 3A and B). These results together show that chronic exposure to excess insulin/glargine induced insulin resistance mainly in liver.

### Chronic exposure to excess insulin (glargine) appears to decrease the number and size of pancreatic islets

**Figure 4:**
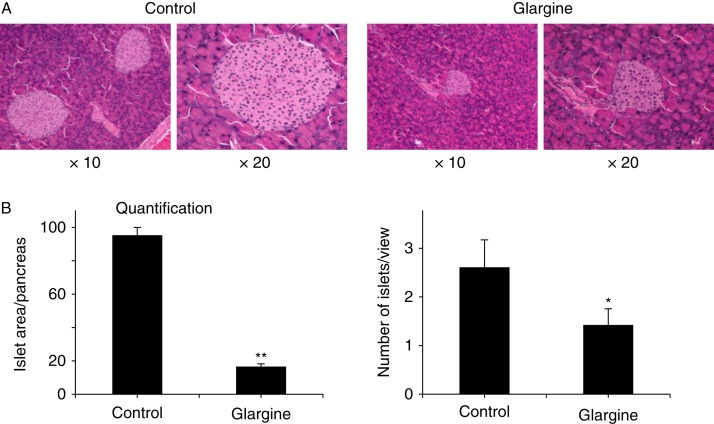
Chronic exposure to excess insulin (glargine) leads to loss of pancreatic islets in animals fed on a chow diet. Pancreas of mice described in Fig. 1 were collected from five mice per group, fixed, sectioned, stained with hematoxylin and eosin, and visualized by microscopy (A). Typical views were presented. The size and number of islets were quantified and analyzed (B). Results represent mean±s.d. of six to eight mice per group. *P<0.05 vs control. **P<0.01 vs control. Full colour version of this figure available via http://dx.doi.org/10.1530/JOE-14-0117.

To determine the effect of chronic exposure to excess insulin (glargine) on pancreatic β-cells, pancreatic islets were examined by histological evaluation. As shown in Fig. 4, both the number and the size of pancreatic islets were dramatically decreased by the treatment with glargine. These results show that chronic exposure to excess insulin appears to reduce pancreatic islet mass.

### Chronic exposure to excess insulin induces ectopic fat accumulation in liver

**Figure 5:**
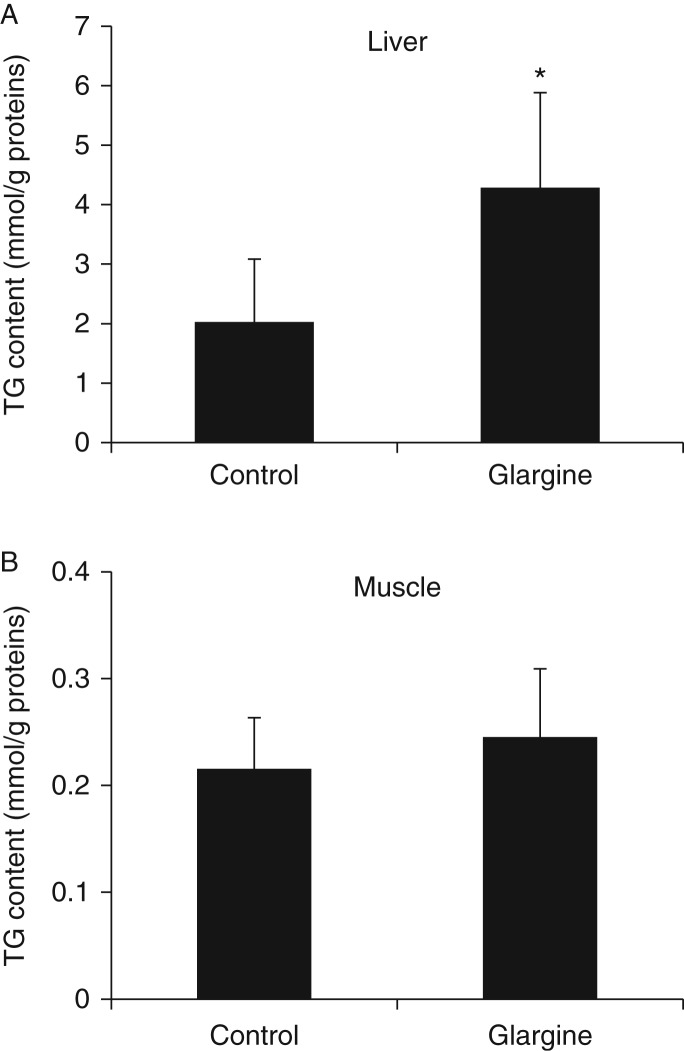
Chronic exposure to excess insulin (glargine) leads to ectopic fat accumulation in liver in mice fed on a chow diet. Triglyceride (TG) content in the liver (A) and muscle (B) of mice described in Fig. 1 was examined as detailed in ‘Materials and methods’. Results represent mean±s.d. of six to eight mice per group. *P<0.05 vs control.

As ectopic fat accumulation is known to be associated with insulin resistance as reviewed previously (Cao *et al*. 2011), TG level was determined in liver and gastrocnemius. As shown in Fig. 5, TG content was significantly enhanced by glargine in liver but not in gastrocnemius. Similarly, treatment with glargine increased the expression of the key lipogenic gene, fatty acid synthase (*Fas*), obviously in liver but not in gastrocnemius (Table 2). The effect of glargine on the expression of genes involved in fat oxidation appeared to be different between liver and gastrocnemius. Specifically, expression of the long-chain acyl CoA dehydrogenase (*Acadl*) gene was decreased by glargine in muscle but not in liver (Table 2). These results together show that chronic exposure to excess insulin (glargine) increases ectopic fat accumulation in liver.

### Chronic exposure to excess insulin (glargine) induces oxidative stress in liver and pancreas

**Figure 6:**
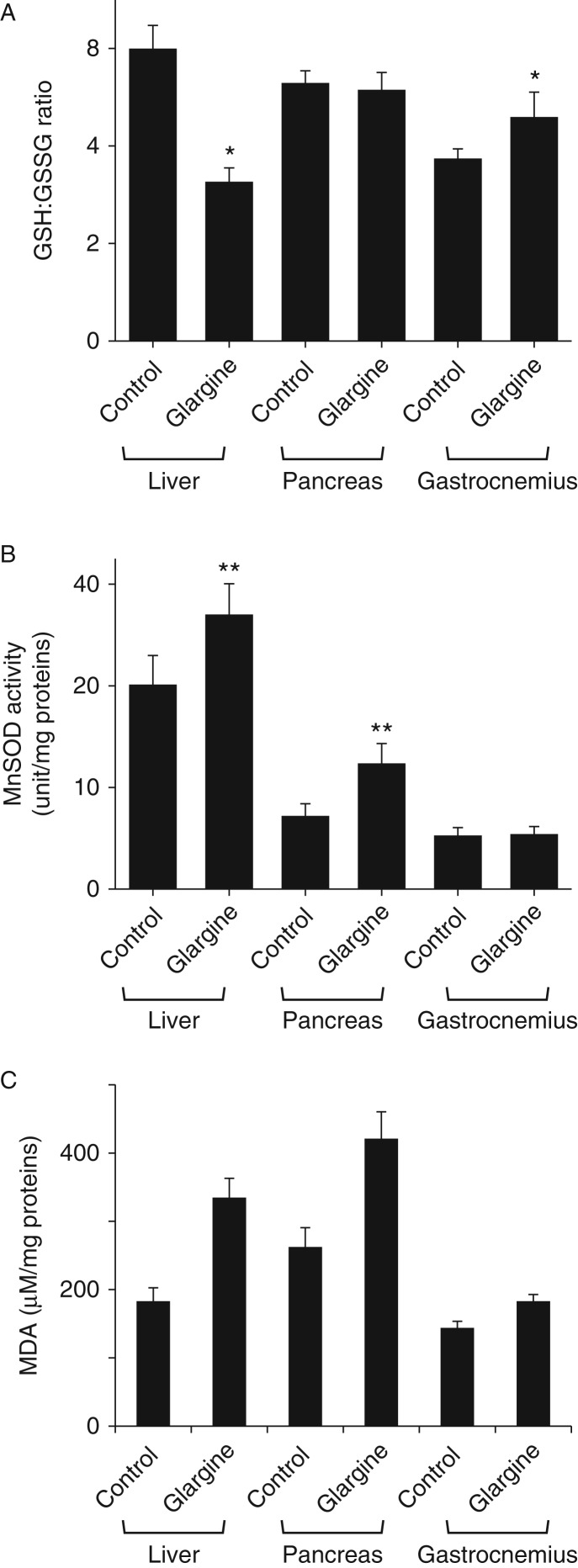
Chronic exposure to excess insulin (glargine) leads to oxidative stress in liver and pancreas in mice fed on a chow diet. GSH:GSSG ratio (A), MnSOD activity (B), and malondialdehyde (MDA) (C) in liver, pancreas, and gastrocnemius of mice described in Fig. 1 were measured and normalized to protein levels of the same samples as detailed in ‘Materials and methods’. Results represent mean±s.d. of six animals per group. *P<0.05 vs control. **P<0.01 vs control.

It is known that oxidative stress plays a critical role in induction of insulin resistance and tissue damage as reviewed previously (Cao *et al*. 2011). Thus, oxidative stress level was examined in liver, gastrocnemius, and pancreas. As shown in Fig. 6A, the GSH:GSSG ratio was decreased by treatment with glargine in liver but not in gastrocnemius and pancreas. MnSOD activity was dramatically increased in both liver and pancreas but not in gastrocnemius (Fig. 6B). To investigate the mechanism by which the mass of pancreatic islets was decreased by glargine, the level of oxidized lipids was determined. As shown in Fig. 6C, glargine treatment increased the level of oxidized lipids (MDA) significantly in liver and pancreas, but not in gastrocnemius. These results together show that chronic exposure to excess insulin induces oxidative stress in liver and pancreas.

### Chronic exposure to excess insulin (glargine) increases cholesterol content in mitochondria of liver and pancreas

**Figure 7:**
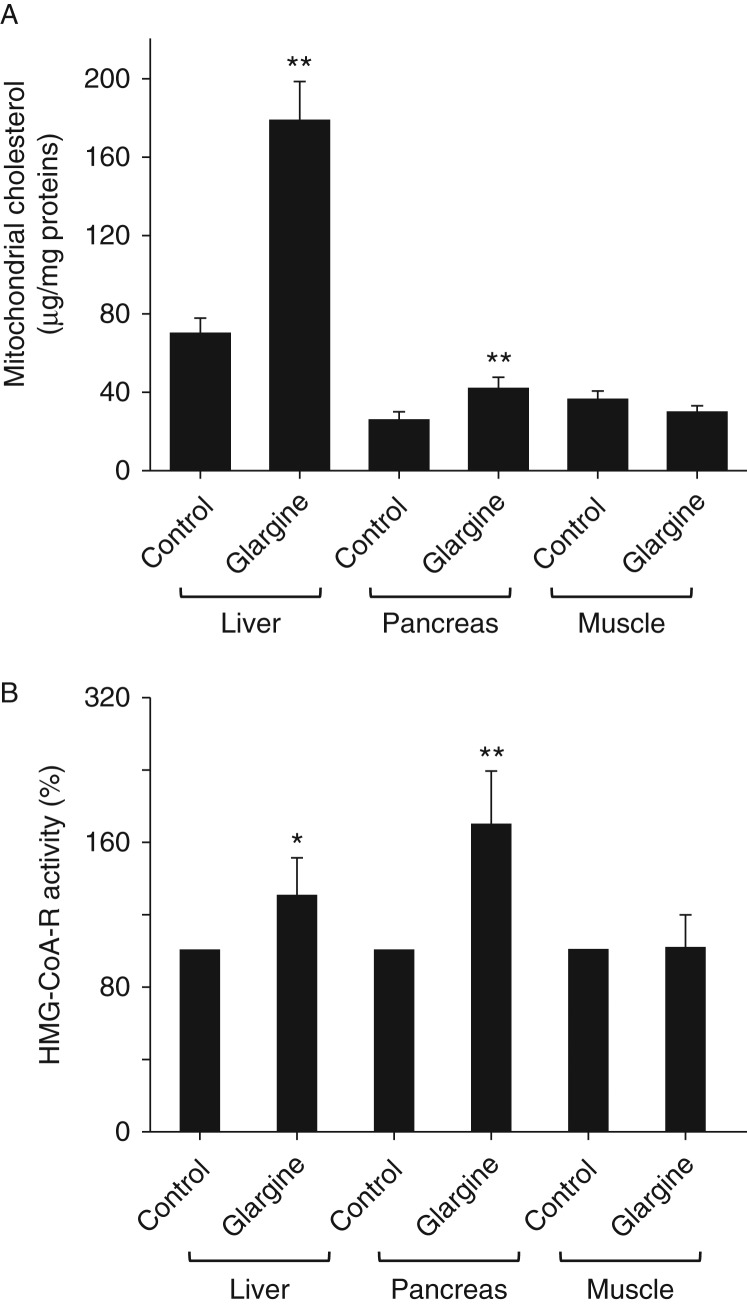
Chronic exposure to excess insulin (glargine) leads to increased cholesterol content in mitochondria of liver and pancreas in mice fed on a chow diet. Mitochondria were freshly isolated from liver, pancreas, and gastrocnemius of the mice described in Fig. 1. Levels of mitochondrial cholesterol (A) and HMG-CoA reductase (HMG-CoA-R) (B) in the noted tissues were measured as detailed in ‘Materials and methods’. Results represent mean±s.d. of six animals per group. *P<0.05 vs control. **P<0.01 vs control.

To further investigate mechanisms of glargine-induced insulin resistance and reduction of pancreatic mass, the cholesterol content in mitochondria of liver, gastrocnemius, and pancreas was quantified. As shown in Fig. 7A, treatment with glargine increased the cholesterol content in mitochondria in liver and pancreas significantly but not in gastrocnemius. The activity of the rate-limiting enzyme of cholesterol synthesis, HMG-CoA reductase, was increased by the treatment with glargine in both liver and pancreas and unaltered in gastrocnemius (Fig. 7B).

### Prolonged exposure to insulin induces oxidative stress in hepatocytes and β-cells

**Figure 8:**
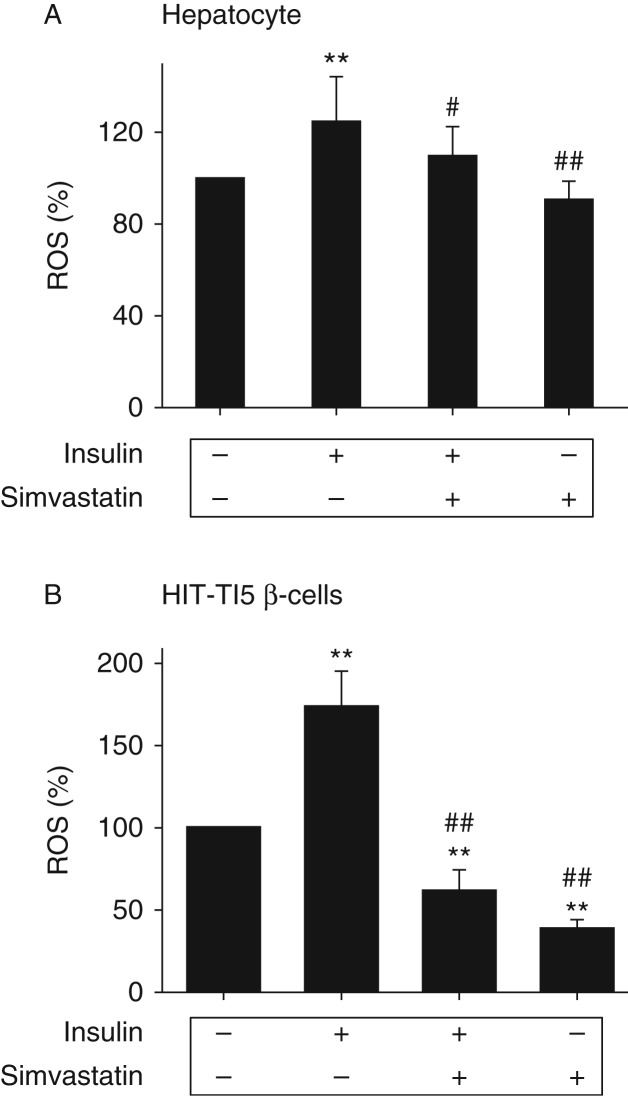
Chronic exposure to excess insulin (glargine) leads to oxidative stress in cultured hepatocytes and β-cells in a cholesterol synthesis-dependent manner. Primary mouse hepatocytes (A) and HIT-T15 β-cells (B) were treated with insulin (5 nM) in the presence or absence of simvastatin (10 μM) for 24 h, followed by the measurements of ROS as detailed in ‘Materials and methods’. Results represent mean±s.e.m. of three independent experiments. **P<0.01 vs no insulin. ^#^ *P*<0.05 vs insulin. ^##^ *P*<0.01 vs insulin.

To further investigate the effect of cholesterol on the insulin-induced oxidative stress, the effect of prolonged exposure to insulin on ROS production and the GSH:GSSG ratio was examined in cultured cells. As shown in Fig. 8A and B, prolonged exposure to insulin increased the level of ROS in both hepatocytes and β-cells, and the increase was prevented by inhibition of cholesterol synthesis with simvastatin. These results together demonstrate that excess exposure to insulin can induce oxidative stress in both liver and pancreas in a cholesterol synthesis-dependent manner.

## Discussion

The exact mechanism by which nutrients cause T2DM has been intensively investigated but remains unestablished. We have recently shown that insulin plays an essential role in mediating the nutrient-induced insulin resistance, a precursor of T2DM (Liu *et al*. 2009*b*, Cao *et al*. 2011, Ning *et al*. 2011). In this study, we tested the hypothesis that chronic exposure to excess insulin alone could cause T2DM and made several novel findings.

First, chronic exposure to excess insulin (glargine) caused insulin resistance, hyperinsulinemia, and relative insulin insufficiency (Fig. 2). These are key features of T2DM. It has long been known that administration of excess amount of insulin to humans can cause hyperglycemia, which has been called the Somogyi phenomenon (Somogyi 1959). It was considered that hypoglycemia caused by the excess insulin leads to increased secretion of gluconeogenic hormones such as growth hormone and elevated gluconeogenesis (Somogyi 1960). However, recent studies did not report any hypoglycemic condition when the blood glucose level was monitored continuously for 24 h in subjects with the Somogyi phenomenon (Hoi-Hansen *et al*. 2005). During this study, we measured the fasting blood glucose level weekly and did not observe any hypoglycemic condition. The close monitoring of animal behaviors did not reveal any sign of hypoglycemia. By contrast, the fasting blood glucose level started to increase on week 1 of the treatment with glargine. We did not observe any increase in plasma level of growth hormone in this study (Table 1). Others did not find the assumed changes in hormones (Gale *et al*. 1980). Therefore, hyperglycemia caused by chronic exposure to excess insulin is not likely to be due to hypoglycemia-induced effects as suggested by Somogyi. Marban *et al*. (1989) have previously shown that hyperinsulinemia (32 times of normal insulin level) induced by transgenic overexpression of the human insulin gene leads to a lean phenotype in mice with severe insulin resistance and normal glycemia. In this study, the fasting blood glucose level started to increase at 1 week after the exposure to excess insulin. The reason for the difference between this study and Marban's study is unclear but most likely to be due to the types of insulin that were used. In Marban's study, the regular human insulin gene was overexpressed. Regular insulin is fast acting and excess amount of it can induce global insulin resistance instantly. Otherwise, the excess regular insulin will cause lethal hypoglycemia. Severe insulin resistance was exactly the same as observed in those animals. In this study, the slow- and long-acting insulin was used. Although the amount of glargine used in this study was large, it did not cause overwhelming hyperinsulinemia and severe global insulin resistance. Instead, the chronic exposure to excess glargine caused only moderate insulin resistance mainly in liver. We believe that in comparison to the overwhelming global insulin resistance induced by transgenic overexpression of the regular insulin, hyperinsulinemia and the associated insulin resistance induced by administration of glargine mimic the hyperinsulinemia and insulin resistance induced by nutrients better. Besides, the insulin in the mice with transgenic overexpression of the insulin gene comes from transgenic overexpression. Thus, those mice will always produce enough insulin to overcome insulin resistance and will not develop insulin deficiency (relative), which is caused by the loss of pancreatic β-cells and is a necessary component of T2DM. Similarly, although insulinoma has been shown to be associated with T2DM in subjects with morbid obesity (Wildbrett *et al*. 1999), subjects with insulinoma mostly do not develop typical T2DM because insulin is secreted in these subjects from a tumor that will always produce enough insulin to overcome insulin resistance. That is why, overall, insulinoma is rarely linked to T2DM (Ishii *et al*. 1993, Kane *et al*. 1993, Lei *et al*. 2007).

Secondly, chronic exposure to excess amount of slow- and long-acting insulin, glargine, induces moderate and tissue-specific insulin resistance. There is no doubt that exposure to excess insulin can cause global insulin resistance through a feedback loop that includes tyrosine-phosphorylated IRS1/2, PI3K, Akt, mTOR, S6K, and serine-phosphorylated IRS1/2 (Accili 2004). However, the insulin resistance observed in this study may not be so simple. First, insulin resistance induced by excess insulin observed in this study occurred mainly in liver as evidenced by the increased level of serine-phosphorylated IRS1 and lack of Akt phosphorylation in response to acute insulin challenge in liver but not in muscle (Fig. 3). Secondly, other key features of insulin resistance such as ectopic fat accumulation and oxidative stress occurred in liver but not in muscle (Figs 5 and 6). Thirdly, the cholesterol content of mitochondria was increased in liver but not in muscle (Fig. 7) and increased mitochondrial cholesterol and cholesterol synthesis induced by the prolonged exposure to insulin are associated with elevated ROS production and oxidative stress in hepatocytes (Fig. 8). Therefore, it appears that the insulin resistance induced by glargine in this study is more likely caused by the increased cholesterol content in mitochondria.

Thirdly, chronic exposure to excess insulin (glargine) decreases the number and size of pancreatic islets. Overt T2DM occurs only in some subjects when a certain amount of pancreatic islets is lost after a long period of insulin resistance and hyperinsulinemia (Accili 2004). The mechanism that causes the loss of pancreatic β-cells in T2DM patients remains unestablished, but it is generally believed that it is associated with oxidative stress (Cao *et al*. 2011). In this study, both the number and size of pancreatic islets were dramatically decreased by the chronic exposure to excess insulin (Fig. 4) although animals were still producing four times more insulin than control animals (Fig. 2), indicating that the reserve of insulin secretion is normally very high and only a small portion of it is normally utilized. Results from this study show that chronic exposure to insulin induced oxidative stress in pancreas (Fig. 6). The increased oxidative stress is linked to elevated cholesterol content in mitochondria (Fig. 7). In cultured β-cells, the increased production of ROS and oxidative stress induced by the prolonged exposure to insulin can be prevented by inhibition of cholesterol synthesis (Fig. 8). Therefore, the loss of pancreatic islets observed in this study was likely to be caused by oxidative stress. These findings have important implications in understanding as to why T2DM patients gradually become insulin dependent after using insulin. It is currently believed that they become more insulin resistant and thus need more insulin to control their blood glucose level after using insulin. However, based on our findings in this study, it is quite possible that those patients may be losing their pancreatic islets faster after using insulin, particularly those who use a relatively large amount of long-acting insulin reagents.

Our findings in this study have a great level of importance because various insulin reagents including long-acting insulin reagents are currently widely used in T2DM patients. Theoretically, it is impossible to use insulin properly if insulin reagents are only injected into patients because insulin level goes up and down in a linear fashion after an injection instead of the pulsatile fashion normally occurring in humans. The loss of pulsatile insulin secretion alone is known to cause insulin resistance (Shanik *et al*. 2008). In other words, the application of insulin reagents via injections as the general practice in T2DM patients now is bound to aggravate insulin resistance in T2DM and increases the usage of insulin, leading to the chronic exposure to excess insulin. The exposure to excess insulin can not only aggravate insulin resistance and lead to loss of endogenous pancreatic islets as observed in this study, but also lead to many other severe consequences. For example, many studies have shown that aggressive control of blood glucose with insulin reagents or stimulators of insulin secretion such as sulfonylureas is associated with increased mortalities due to various cardiovascular disorders or cancer (Eurich *et al*. 2007, Anselmino *et al*. 2008, Currie *et al*. 2009, Baur *et al*. 2011, Buchs & Silverman 2011, La Vecchia 2011, Lebovitz 2011, Nandish *et al*. 2011, Sehra *et al*. 2011, Currie & Johnson 2012). Therefore, the current practice in using insulin reagents in T2DM patients requires more caution and more thorough and long-term evaluations.

## Author contribution statement

X Y and S M performed experiments together and equally contributed to this study. H G and H G performed some evaluations including immunoblottings. L Z, J C, and X L helped writing and proofreading the manuscript. Z L provided advice for planning and carrying out the experiments. W C supervised the whole experiments and wrote the manuscript. Dr W C is the guarantor of this work, had full access to the data, and takes full responsibility for the integrity of data and the accuracy of data analysis.

## Figures and Tables

**Table 1:** Serum levels of cytokines, hormones, and lipids. Results represent mean±s.d. of six to eight mice

	**Control**	**Glargine**
Leptin (ng/ml)	0.69±0.05	0.70±0.09
Adiponectin (μg/ml)	6.15±1.25	6.17±1.54
TNFα (ng/ml)	0.12±0.06	0.13±0.05
Growth hormone (ng/ml)	20.63±7.45	14.94±9.85
Triglyceride (mmol/l)	0.61±0.10	0.65±0.14
Free fatty acids (mmol/l)	0.48±0.12	0.44±0.07
Total cholesterol (μg/ml)	34.38±9.76	44.25±13.82*

Compared with control group, *P<0.05.

**Table 2:** The mRNA levels of genes involved in lipogenesis and fat oxidation. Results represent mean±s.d. of six to eight mice

	**Glargine vs control** (fold)			
Liver	*P* value	Muscle	*P* value
*Cpt1* *α* * (Cpt1* *β* *)* (*Chpt1*)	0.824±0.581	0.580	1.131±0.893	0.755
Acadm	1.542±1.118	0.328	0.565±0.161	0.077
Acadl	1.351±0.850	0.411	0.406±0.179*	0.011
Srebp1 (Srebf1)	1.042±1.158	0.942	0.933±0.371	0.829
Srebp2 (Srebf2)	0.752±0.581	0.539	0.860±0.899	0.838
Fas	2.667±0.919^†^	0.007	1.198±1.178	0.838

Compared with control group, *P<0.05; ^†^ *P*<0.01.
